# Erratum to: The neural correlates of value representation: From single items to bundles

**DOI:** 10.1002/hbm.26473

**Published:** 2023-09-04

Zyuzin, J., Combs, D., Melrose, J., Kodaverdian, N., Leather, C., Carrillo, J. D., Monterosso, J. R., & Brocas, I. (2023). The neural correlates of value representation: From single items to bundles. *Human Brain Mapping*, *44*(4), 1476–1495. https://doi.org/10.1002/hbm.26137

In the published version of the article, James Melrose, Niree Kodaverdian, Calvin Leather, and Juan D. Carrillo were missing from the author list. The corrected authorship appears below. The online version of this article has been corrected accordingly.

Jekaterina Zyuzin^1^, Dalton Combs^1^, James Melrose^2^, Niree Kodaverdian^3^, Calvin Leather^2^, Juan D. Carrillo^2^, John R. Monterosso^4^, Isabelle Brocas^2^

^1^University of Southern California, Los Angeles, California, USA.

^2^Department of Economics, University of Southern California, Los Angeles, California, USA.

^3^Argyros School of Business and Economics, Chapman University, Orange, California, USA.

^4^Department of Psychology, University of Southern California, Los Angeles, California, USA.

